# Defining the wheat microbiome: Towards microbiome-facilitated crop production

**DOI:** 10.1016/j.csbj.2021.01.045

**Published:** 2021-02-09

**Authors:** Vanessa N. Kavamura, Rodrigo Mendes, Adnane Bargaz, Tim H. Mauchline

**Affiliations:** a Sustainable Agriculture Sciences, Rothamsted Research, Harpenden, Hertfordshire, UK; b Laboratory of Environmental Microbiology, Embrapa Environment, Jaguariúna, SP, Brazil; c Agrobiosciences, Mohammed VI Polytechnic University, Benguerir, Morocco

**Keywords:** Wheat, Rhizosphere, Microbiome, Sustainable intensification

## Abstract

Wheat is one of the world’s most important crops, but its production relies heavily on agrochemical inputs which can be harmful to the environment when used excessively. It is well known that a multitude of microbes interact with eukaryotic organisms, including plants, and the sum of microbes and their functions associated with a given host is termed the microbiome. Plant-microbe interactions can be beneficial, neutral or harmful to the host plant. Over the last decade, with the development of next generation DNA sequencing technology, our understanding of the plant microbiome structure has dramatically increased. Considering that defining the wheat microbiome is key to leverage crop production in a sustainable way, here we describe how different factors drive microbiome assembly in wheat, including crop management, edaphic-environmental conditions and host selection. In addition, we highlight the benefits to take a multidisciplinary approach to define and explore the wheat core microbiome to generate solutions based on microbial (synthetic) communities or single inoculants. Advances in plant microbiome research will facilitate the development of microbial strategies to guarantee a sustainable intensification of crop production.

## Graphical abstract

**Figure:**
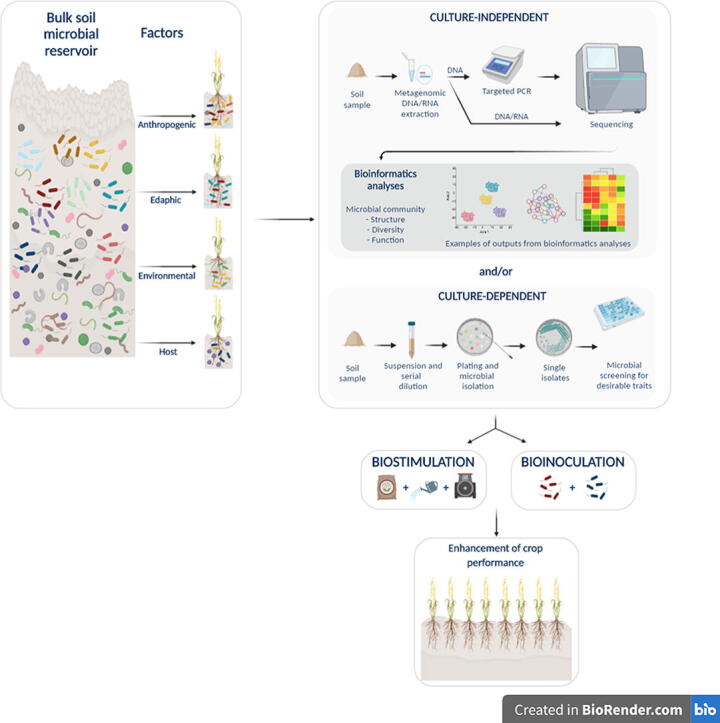

## 1 Introduction – wheat and agricultural intensification on a fast-growing world

Wheat was one of the first domesticated crops, between 7000 and 9000 BCE, and has undergone a process of expansion to global cultivation [1]. Bread wheat, *Triticum aestivum* L., is the most widely cultivated species, with more than 20,000 known varieties. It is one of the most important crops worldwide, occupying 17 percent of the total cultivated land in the world and providing the staple food for 35 percent of the world’s population [2]. Between 10,000 and 4000 years ago people began growing food, which led to the domestication of wild crops and the emergence of agriculture [3]. Agricultural progress has supported population growth, which globally now is estimated to be 7.7 billion [4]. Wheat is a major world crop, but to meet the calorie requirement of an increasing world population, an 11% increase in wheat production is required by 2026 with just a 1.8% increase in cultivation area [5]. Furthermore, it is estimated that by 2050, population size will exceed 9.7 billion [4]. A process of sustainable agricultural intensification must be implemented to make these crop productivity gains [6], [7] which will result in enhanced yield through increases in crop tolerance to biotic and abiotic stresses, improved nutrient use efficiency as well as the development of new bio-fertilizers [8], [9]. It is well known that plants are colonized by microorganisms which can be beneficial to the host, and the potential of microbes to contribute to these sustainability goals has gained traction over the last years. A better understanding of patterns of microbiome assemblage is of fundamental importance as a prerequisite for the use of the microbiome in sustainable agriculture. In this review, we focus on factors driving the wheat microbiome assembly. Additionally, we highlight the gaps that need to be addressed towards a microbially-assisted sustainable intensification of wheat production. Finally, we briefly discuss the use of the microbiome as a source of microbial inoculants, through the application of synthetic communities (bioinoculants) and/or via optimization of agricultural practices to stimulate the beneficial indigenous microbial communities (biostimulation).

## 2 Factors affecting wheat microbiome structure and diversity

**Fig. 1:**
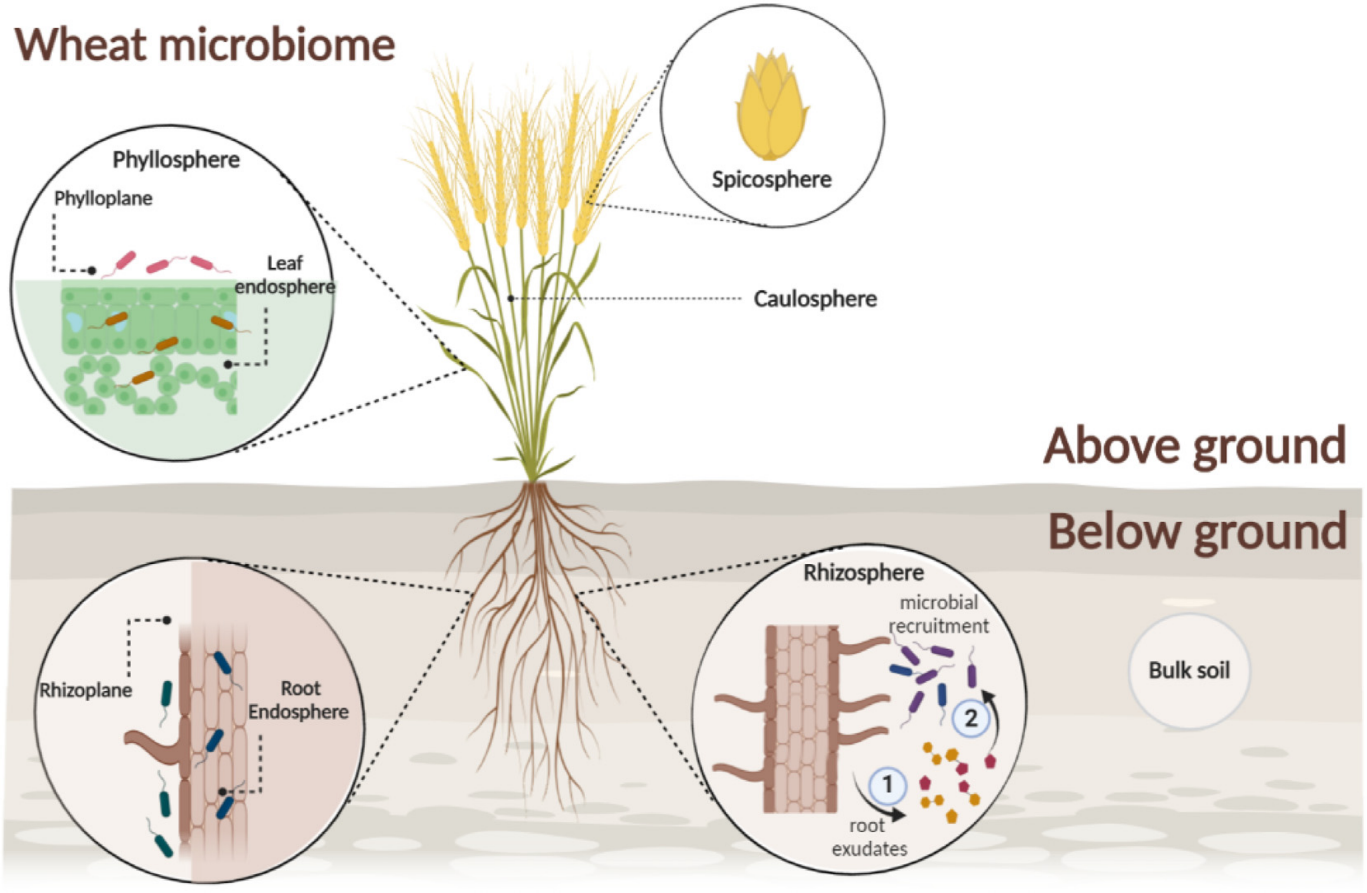
The wheat microbiome is divided into above- and below-ground sections. The below-ground compartments are the rhizosphere and rhizoplane. The above-ground compartment is known as the phyllosphere, and subdivisions of this include the caulosphere and “*spicosphere”*, with a detail of a spikelet. Created with BioRender.com.

The advent of high throughput DNA sequencing technologies has facilitated amplicon sequencing-based research, metagenomics and metatranscriptomics to determine the composition and functions of microbial communities associated with different crops. This has allowed the understanding of how different factors affect microbial communities associated with host plants in unprecedented detail in different niches in and around the host plant. Broadly speaking these can be divided into above-ground and below-ground niches. The phyllosphere [10] refers to the above-ground parts of the plants, and most commonly to the leaves. The above-ground compartments comprise the leaves, stems (caulosphere) [11], seeds and spikes or heads. In addition, we propose the term “*spicosphere*” as the niche comprised of wheat spikes, as it is an important reservoir for pathogenic and beneficial microorganisms living inside and on the surfaces of the rachis and spikelets (comprised of lemma, palea, glume, floret, awn and grain). Below-ground compartments can be divided into the rhizosphere [12], the soil influenced by the host plant largely through root exudation, and the rhizoplane [13], the surface of the root. In addition, microbes can reside within intercellular spaces (endosphere), either in above- or below-ground tissues as endophytes [14], [15] (Fig. 1). Additionally, spermosphere is the term related to the dynamic zone surrounding germinating seeds [16], [17].

In addition to niche, many factors have been evaluated either alone or in combination to determine their influence on the wheat microbiome (Table 1). These include factors which are dependent on human interference (**anthropogenic**)**,** soil-related factors (**edaphic**)**, environmental**, which are related to natural conditions and **host** factors which are dependent on the plant species.

**Table 1:** Evaluation of factors to determine their influence on the wheat microbiome.

Type	Factor	Reference
**Anthropogenic**	Exogenous compounds (fungicide)	[18], [19]
	Exogenous compounds (glyphosate)	[20]
	Exogenous compounds (insecticides)	[21]
	Exogenous compounds (phosphine fumigation of stored wheat grains)	[22]
	Exogenous compounds (plastic mulch film residues)	[23]
	Fertilization	[24], [25], [26], [27], [28], [29], [30], [31], [32], [33]
	Inoculation of biocontrol agent	[26], [34], [35]
	Land use	[36], [37], [38]
	Management type	[39], [40], [41]
	Overhead irrigation	[42]
	Rotation	[43], [44], [45], [46], [47], [48]
	Tillage	[40], [41], [44], [48], [49]

**Edaphic**	Soil depth	[50], [51]
	Soil history	[52]
	Soil physicochemical characteristics	[24], [29], [50], [53], [54], [55]
	Soil type	[32], [35], [56]

**Environmental**	Abiotic stresses (e.g. drought, humidity and temperature)	[42], [52], [57], [58], [59], [60]
	Biotic stresses (pathogens, weed)	[34], [35], [41], [61], [62], [63], [64], [65], [66]
	Geographical location	[32], [38], [43], [49], [53], [64], [67], [68], [69], [70]
	Growing season	[38], [41], [56], [63], [67]

**Host**	Breeding and domestication	[37], [71], [72], [73], [74], [75], [76]
	Genotype	[32], [33], [37], [43], [52], [55], [58], [60], [69], [70], [77], [78]
	Growth stage	[25], [27], [30], [34], [35], [39], [43], [60], [64], [70]
	Leaf position	[70]
	Niche	[26], [36], [38], [43], [44], [47], [49], [53], [54], [58], [67]
	Organs/Tissues	[24], [30], [35], [39], [58], [64], [79], [80]
	Plant hormones	[81], [82]

In the following sections, we focus on the different factors that affect the wheat microbiome structure, diversity and function. It is important to note that the factors discussed here are not exhaustive and exclusive, meaning there can be interactions of different factors accounting for changes in the wheat microbiome.

### 2.1 Anthropogenic factors driving microbiome assembly

#### 2.1.1 Exogenous compounds

Current conventional agriculture relies heavily on the use of exogenous compounds which can be environmentally damaging as well as threatening to human health [83], [84]. These include the use of agrochemicals such as fertilizers, fungicides, insecticides and pesticides. However, research into the effect of the treatment of wheat seeds with neonicotinoid insecticides has revealed that they do not negatively impact wheat rhizosphere microbial communities [21]. Similarly, the repeated pre-harvest application of glyphosate, the most widely used herbicide [85], had minimal impacts on soil and rhizosphere bacteria of wheat, with a small number of copiotrophic taxa benefiting from dying roots in the soil [20]. However, it’s important to highlight that in-field applications of glyphosate can differ, thus in the later, the authors conducted a 3-year experiment in which glyphosate was applied at the end of six weeks, to simulate a pre-harvest application. Safer alternatives to these compounds could be the use of microbial-based natural products. The use of microorganisms as biological control agents is an environmentally benign alternative to pesticides [86], though a better understanding of these interactions is required to develop sustainable strategies to aid the establishment and persistence of beneficial microbes in agricultural systems. Besides, it is crucial to understand their impacts on indigenous soil microbial communities, given their role in the functioning of ecosystems. For example, Araujo et al. [34], [35] challenged soils infected with *Rhizoctonia solani and Pythium* sp. with biocontrol agents (*Paenibacillus fulvissimus* and *Streptomyces* spp.) to monitor changes in wheat microbial communities. Biocontrol isolates were able to modulate the endosphere and rhizosphere microbiomes, with generally low impact on indigenous microbial communities, as well as with a decrease in root disease and positive impacts on plant growth. The use of both low-density polyethylene (LDPE) and biodegradable plastic mulch films to increase crop productivity has been evaluated [23] and the authors observed a significant effect of the residues on rhizosphere bacterial community composition and structure and volatiles emission, suggesting future efforts should concentrate at developing experiments to increase the understanding of these compounds on agroecosystems.

The impact of fertilizers on microbial communities is well studied. Application of high levels of inorganic nitrogen fertilizers reduced bacterial richness and diversity, leading to a less stable bacterial community structure, and this was exacerbated with increased crop maturity. Members of Acidobacteria and Planctomycetes were significantly depleted in treatments receiving inorganic N and 16S rRNA gene-predicted functional structure was also impacted [27]. In another study the use of organic amendments such as biochar and manure were compared to the use of mineral fertilization on above (spikelet) and belowground (rhizosphere and root) bacterial communities, with significant changes in their structure and diversity [24]. In addition, Chen et al. [25] found that nitrogen fertilization affected rhizosphere bacterial communities isolated from wheat plants during tillering but not during jointing and ripening.

#### 2.1.2 Agricultural practices

Agricultural practices such as tillage and crop rotation can have detrimental effects on the environment, such as emissions of greenhouse gases (GHGs) [87]. No-tillage practices have been shown to reduced global warming potential when compared to conventional tillage [88]. The effect of tillage is stronger in the bulk soil than rhizosphere [49]. Similar findings were observed by Lupwayi et al. [44], in which the effect of tillage was more prominent in bulk soil than rhizosphere with significant decrease in bacterial diversity in the bulk soil.

Conventionally-tilled wheat monoculture and wheat-soybean rotation resulted in a lower bacterial diversity compared with the no-till treatment [48]. Hartman et al. [40] investigated the impact of common cropping practices (management type and tillage intensities) on bacterial and fungal communities in winter wheat. Root bacterial communities (rhizoplane or endosphere) were primarily affected by management type (conventional vs organic), whereas fungal communities were generally influenced by changes in tillage intensity.

Long-term monoculture can change soil properties, affecting bacterial diversity and this has been demonstrated [45]. Although they used maize monoculture, they were able to show that humus content was lower when compared to maize-wheat rotation, suggesting that lower concentrations of humus could decrease the amount of available nutrients for plant growth and decrease microbial richness. Some positive impacts of rotation of sunflower with wheat and maize on bacterial communities were observed, which could potentially alter plant productivity in agricultural systems [46].

In a study conducted using samples from the Highfield experiment at the Rothamsted Research farm in Harpenden, Hertfordshire, UK [89], conversion of grassland to an arable system resulted in a significant reduction in the abundance of OTUs assigned to specific bacterial taxa [36]. When comparing wheat grown in arable and forest soil, Rossmann et al. [37] observed that the soil type had major impacts on bacterial and cercozoan rhizosphere communities and less influence on fungal community composition.

### 2.2 Edaphic conditions driving microbiome assembly

It is well known that differences in soil physical and chemical properties drive microbiome community structure in wheat. Amadou et al. [24] observed that the amendment of soil with biochar and manure as well as the addition of inorganic mineral fertilizers changed soil properties, in particular NH_4_^+^ content, and these impacted above (spikelet) and belowground (rhizosphere and root) bacterial community structure. Organic amendments can improve water retention and are associated with increased acid phosphatase, β-1,4-*N*-acetyl-glucosaminidase and phenol oxidase activity, whereas inorganic fertilizers lower the pH, increasing nutrient assimilability. Changes in chemical properties of rhizosphere soil, such as pH and nutrient availability which impact bacterial communities can also be attributed to root exudates [53]. Soil pH is the main driver of microbial community structure including archaeal, bacterial and fungal members [53], [54]. Soil texture has also been shown to be important in structuring microbial communities [56].

Most soil microbial community structure studies have concentrated on the topsoil. However, Schlatter et al. [50] and Uksa et al. [51] have characterized the composition and diversity of bacterial communities across a wide range of soil depths. Both observed that Proteobacteriota are enriched in the topsoil, though the former also observed that Acidobacteria were more abundant at 10 cm, presumably because of soil acidification from fertilizer application. In addition, Uksa et al. [51] also observed that Firmicutes and Bacteroidota taxa were enriched in the subsoil.

### 2.3 Environmental factors driving microbiome assembly

#### 2.3.1 Abiotic factors

In addition to soil properties, several abiotic factors can affect microbial communities. Latz et al. [58] observed location-dependent effects (in the glasshouse and outside the glasshouse) on wheat microbiome composition, which were likely a result from differences in the environmental conditions (temperature, humidity and precipitation). Water is one of the most limiting factors for plant development and agricultural losses due to drought are quite substantial. Azarbad et al. [52] investigated the influence of soil water stress history, wheat genotypes with differences in their drought tolerance, and short-term decrease in soil water content on microbial communities of wheat. Soil history, in this case, was soil from two fields which have been subjected to irrigation and no irrigation for almost 40 years. It was found that water regime was the main driver of bacterial and fungal community structure in the rhizosphere and root samples of wheat. Stromberger et al. [60] investigated the effect of different irrigation regimes on bacterial communities and observed an enrichment of 1-aminocyclopropane-1-carboxylic acid (ACC) deaminase bacteria in the rhizosphere of a drought tolerant cultivar, indicating that it either produces more ACC and ethylene or is more effective in recruiting ACC deaminase expressing bacteria into this niche. Mavrodi et al. [42] conducted a three-year field study on wheat grown in irrigated and non-irrigated plots to assess the effect of soil water status on bacterial communities. A decrease in the production of the antibiotic phenazine-1-carboxylic acid (PCA) and associated PCA producers (Phz+) *Pseudomonas* in the rhizosphere of irrigated plants was observed. They hypothesised that an increase in soil moisture perturbs interactions within the rhizosphere microbiome, altering the root exudation and soil properties.

#### 2.3.2 Biotic factors

Biotic factors such as the presence of pathogens is another deterministic factor. Wheat residues can determine the epidemiology of Septoria tritici blotch as they support the growth of the causal fungal agent *Zymoseptoria tritici* [63]. Their results show that pathogen infection dynamically changes bacterial and fungal interactions. In addition, it has become evident that soils inoculated with pathogens can become suppressive over time to specific pathogens [66]. Enrichment and activation of bespoke groups of microorganisms in soil can lead to microbial suppression of pathogens, however, the factors which contribute to the development of these systems are not yet fully understood [90], [91]. Yin et al. [66] showed that *Chryseobacterium* and *Pseudomonas* became more prevalent in the rhizosphere over time after soil inoculation with *Rhizoctonia solani.* These strains exhibited inhibitory activities against the fungus *in vitro* or reduced the infection in soils, indicating that they might play a role in the transition of conduciveness to suppressiveness. Hayden et al. [61] used a metatranscriptomics approach to characterize the active members and functions of the wheat rhizosphere microbiome in suppressive and conducive soil conditions to *Rhizoctonia solani.* They described the gene expression in the tri-trophic interaction and propose that this information can be used to direct management options to promote beneficial rhizosphere microbiota colonization and activity to reduce pathogen infection.

Similar to the gut microbiome, which is known to play an important role in host health [92], the microbiome of plants helps them tolerate biotic and abiotic stresses [93]. Thus, understanding the plant-microbiome interactions can be used to manage abiotic and/or biotic stresses. In addition, host defense mechanisms have an important role in structuring microbial communities [94], [95]. Teixeira et al. [95] proposed that the microbiome can protect the host against pathogens, directly via suppression with secondary metabolite production or through competition for resources; as well as indirectly, via the stimulation of the host’s immune system. In other cases, pathogens have evolved mechanisms to overcome the immune defense. For example, the wheat pathogen *Zymoseptoria tritici* has been shown to induce systemic host susceptibility through altered plant metabolism and microbial community structure, making it more vulnerable to infection [65].

There are several other environmental factors that can contribute to differences in microbiome structure, diversity and function. Biogeographic studies aim to evaluate the distributions of soil microbial diversity, composition and functions over space and time from regional to global scales [96]. Fan et al. [53] studied nine wheat fields distributed across 800,000 km^2^ to study the influence of geographical distance on bacterial communities from loosely and tightly bound rhizosphere soil, suggesting that geographic distance was the main driver of community distribution. Schlatter et al. [38] explored bacterial and fungal communities of wheat grown in soil from four distinct locations, observing significant effects on the structure and composition of microbial communities which could be linked with differences in soil properties as previously discussed.

Finally, seasonal changes can also account for differences in wheat microbiome. Schlatter et al. [56] observed significant effects of the growing season on bacterial and fungal community composition, however, richness and diversity were not affected.

### 2.4 Host microbiome selection

#### 2.4.1 Niche, plant compartment and seed load

Niche plays an important role in shaping microbial communities. The root acts as a physical barrier and a subset of these bacteria can colonize the endosphere [36], [97]. In addition to the bulk soil-derived microbial colonization of the plant host, the microbial seed load is also a source of microbes capable of colonizing the developing plant. Kavamura et al. [36] found using an embryo excision-based approach, that the seed-borne bacterial community was important for shaping the endosphere of wheat when plants were cultured in soil that was not adapted for wheat, whereas this was not the case for the rhizosphere community. In addition, Cordero et al. [67] demonstrated that when growing the same plant species on agricultural soils, variations between the endosphere and rhizosphere microbiome were observed, suggesting that the root microbiome is under a greater degree of host control. Specific phyla have been identified to be associated with different wheat compartments, with Proteobacteriota being the most abundant in the root endosphere, whereas Firmicutes and Actinobacteriota were more prevalent in the endosphere of leaves [30]. To identify which factors contributed the most in shaping the fungal endosphere microbiome of different wheat compartments (roots, leaves and seeds), Latz et al. [58] analyzed ITS amplicon sequencing of wheat grown indoors and outdoors and concluded that environmental factors were more important for phyllosphere than rhizosphere and that airborne fungi are the main source of leaf and seed microbes. Donn et al. [43] performed a cross-year analysis of bacterial communities in an intensive wheat cropping system and observed changes over time in rhizosphere communities and those differences were not observed for bulk soil samples, suggesting they were plant instead of seasonally driven. In comparison to the bulk soil, rhizosphere microbial communities are less complex and more stable as demonstrated by co-occurrence networks [54]. In a more complete and recent study, Xiong et al. [47] demonstrated the strong selection imposed by the host, showing a decrease in diversity and complexity of bacterial communities from bulk soil > rhizosphere soil > rhizoplane > phylloplane > root endosphere > leaf endosphere. Rhizosphere is the most studied niche, followed by the phyllosphere. The microbiome of wheat spikes is less well documented; however, this niche is important as some pathogens infect the spikes, such as *Fusarium graminearum* and *Magnaporthe oryzae* pv. *Triticum* (MoT), causal agents of Fusarium head blight (FHB) and wheat blast, respectively. However, it is known that bacterial diversity is lower in spikes than in the rhizosphere [24]. In addition, Rojas et al. [64] observed that when wheat is infected by *Fusarium*, a shift in fungal endophytic community colonization dynamics occurs. Furthermore, some genera (*Cladosporium, Itersonillia* and *Holtermanniella*) were found to outcompete the pathogen, preventing the development of the disease. The bacterial endophytes of wheat endosperm, germ, coleoptiles as well as roots and leaves were studied by Kuźniar et al. [80]. They found several beneficial bacteria and *Pseudomonas* spp. was the only genus that was detected in all samples. Vertical transmission of the wheat microbiome was assessed and taxa belonging to *Erwinia,* Rhizobiales and fungal genus *Emericella* might be vertically transmitted from seeds to sprouts [79].

#### 2.4.2 Plant domestication, breeding and wheat genotype

The introduction of reduced height (*Rht*) dwarfing genes into modern wheat cultivars during the Green Revolution resulted in plants with increased yields when cultured with high fertilization application, without productivity losses caused by lodging [98]. Consistent and continuing reductions in height with increases in yield were achieved worldwide [99]. Effectuated by breeding efforts, modern crops have diverged genetically and phenotypically from their wild relatives. Selection for improved wheat varieties may have resulted in changes to root architecture and physiology, which in turn might have affected microbial communities [100], [101]. Wheat root-associated microbiomes have dramatically changed through a transect of breeding history [73]. Differential recruitment of bacterial communities in tall and semi-dwarf wheat cultivars suggest breeding might have affected the ability of wheat to select and sustain a complex bacterial community in the rhizosphere [72], negatively impacting the ability of modern plants to interact with plant growth-promoting rhizobacteria [76]. Similar findings were reported by Rossmann et al. [37], where the effect of wheat domestication on bacterial, fungal, and communities of cercozoa was evaluated. Both domestication and breeding affected network topology, with microbial co-occurrence networks from landraces and tall wheat cultivars being more connected, suggesting a reduced functional redundancy in the root microbiome of modern cultivars. Fungal endophyte communities in wild wheat are richer and more diverse than in cultivated wheat, representing a greater reservoir of potentially beneficial endophytes as a higher proportion of differentially abundant taxa was found [74]. The consequences of plant breeding for the associated microbiome are not yet fully understood, however, it has been proposed that domestication has disrupted selective processes in the assembly of the wheat microbiome [71]. A synthetic hybrid hexaploid wheat was created to recapitulate the breeding history of wheat, suggesting that the D genome from *Ae. tauschii* (diploid) strongly select for Glomeromycetes and Nematoda. Besides, the ratio of eukaryotes to prokaryotes remains the same, likely due to a protective mechanism against soil-borne fungal diseases in wheat, which might be intrinsic to the wheat genome [75].

The effect of different wheat genotypes has been thoroughly investigated [32], [33], [43], [52], [55], [58], [60], [69], [70], [77], [78] and those differences could be attributed to the differential root exudate chemistry [60], [69], [78] and disease susceptibility [70], [77]. The use of genome-wide association studies (GWAS) will likely improve our understanding of the genetic basis of microbiome selection by host plants [58].

#### 2.4.3 Developmental stages

The plant microbiome structure dynamically changes over time from seed to the flowering stage. Donn et al. [43] demonstrated the evolution of bacterial communities within the rhizosphere, with an increased diversity with plant age and senescence. It appears that growth stage has a stronger influence on bacterial communities than on fungal community composition [25]. Araujo et al. [34] observed that the diversity of bacterial genera increased over time, with some bacterial genera dominating the initial stages, such as *Agrobacterium*, *Bacillus*, *Flavobacterium*, *Rhizobium*, and *Rhodoplanes*, whereas other genera increased in the later stages, mainly *Actinoallomurus*, *Aminobacter* and *Mycobacterium*. Regarding fungal communities, *Alternaria*, *Fusarium*/*Gibberella*, and *Lewia* were common in the early stage and *Exophiala* at 12 weeks. The same trend in increased diversity over time was observed for endosphere communities. Gdanetz and Trail [39] observed an increase in both bacterial and fungal endosphere community diversity over time (vegetative, flowering and seed development stages) which could be explained by the ecological succession within the plant microbiome or a reflection of responses to metabolites produced by plant maturation. Sapkota et al. [70] studied the spatiotemporal variation in fungal communities within the wheat canopy at different growth stages, describing key fungal species in the phyllosphere and a general increase over time. However, Kavamura et al. [27] found that when comparing contrasting fertilization regimes, a reduction in bacterial richness was observed over time in the rhizosphere. It was also found that taxonomical diversity remained stable over time following high N application, although, a reduction was seen when N supply was suboptimal. In addition, Robinson et al. [30] when studying the root and leaf endosphere, a reduction in bacterial species richness with increased plant maturity regardless of fertilization regime was detected. As such, the relationship between microbial community composition and growth stage is complicated as it is influenced by many factors.

## 3 Core wheat bacterial communities

We have described the major drivers of microbiome structure in wheat. In addition, it is important to consider the core microbiome, members being consistent features of a dataset that are hypothesized to reflect underlying functional relationships with the host [102]. Different approaches have been used to determine the core microbiome of plants such as the use of a theoretical framework [103], abundance-occupancy distribution [102], microbiome package in R [32], [104], network analyses [105], DESeq2 [38], QIIME 2 [37], [106], [107]. Although the term “core microbiome” has been widely used, there is disagreement surrounding its definition and to the method that should be deployed to define the core microbes which are associated with a given host [108].

**Fig. 2:**
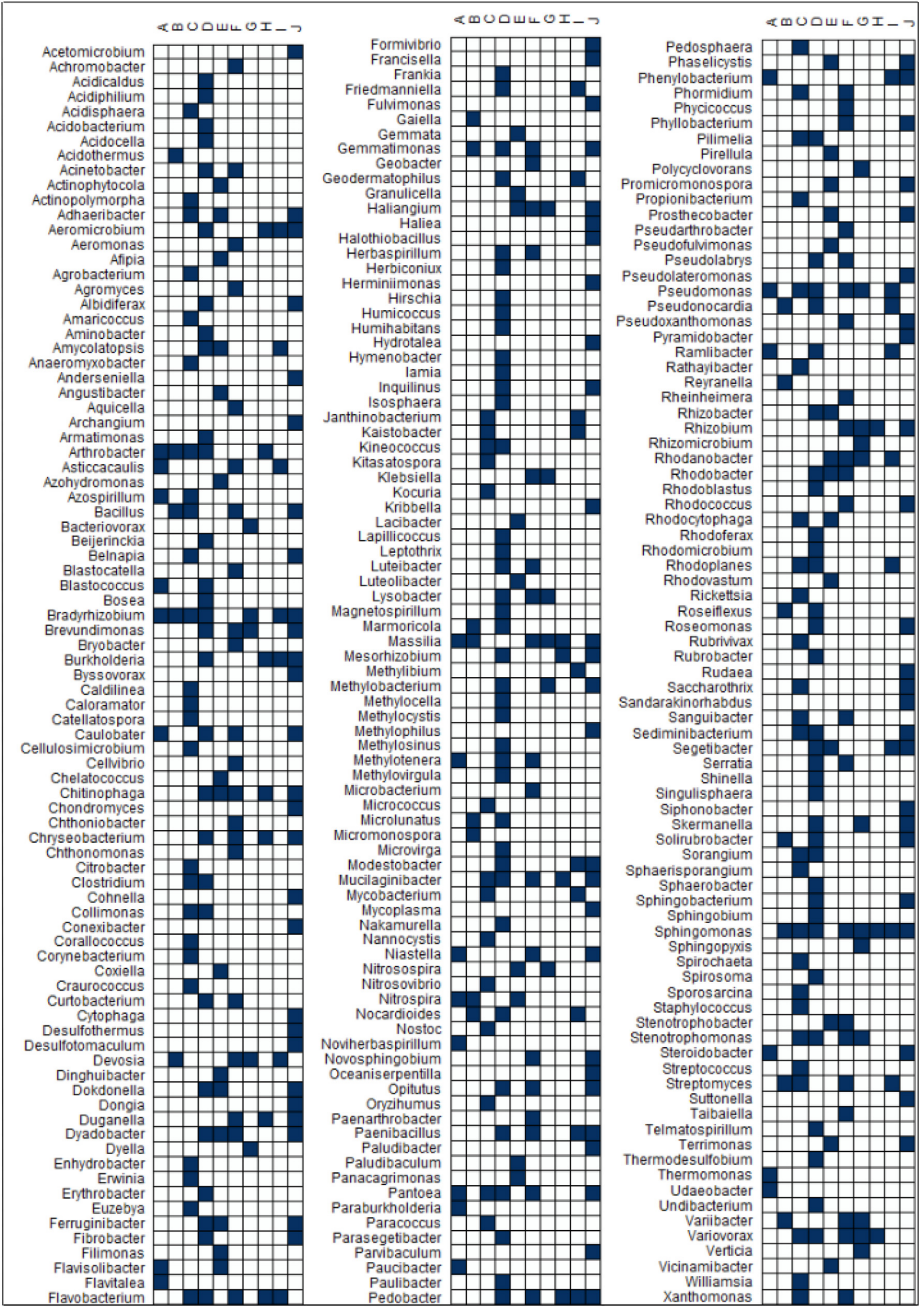
Correlation plot showing 256 bacterial genera commonly associated to wheat from ten studies (A-J) (A-Simonin et al. [32]; B- Rossmann et al. [37]; C- Araujo et al. [35]; D- Mahoney et al. [69]; E- Kavamura et al. [27]; F- Kavamura et al. [36]; G- Kavamura et al. [72]; H- Schlatter et al. [38]; I- Schlatter et al. [56]; J- Mavrodi et al. [42]). Studies A and B determined the core microbiome using R microbiome package and QIIME, respectively. Studies C and D used networks to identify keystone taxa. Studies E-J identified differentially abundant taxa using DESeq2.

Attempts to define the core microbiome of wheat have utilized large datasets [38]. One study identified a core microbiome of 30 bacterial, 24 fungal and 10 taxa assigned to protists by utilizing data from three wheat genotypes grown in eight contrasting soils from Europe and Africa [32]. In another study, Rossmann et al. [37] identified 22 bacterial and 13 fungal taxa and 3 taxa assigned to protists corresponding to the core microbiome of modern wheat cultivars. However, only four bacterial genera (*Arthrobacter, Bradyrhizobium, Massilia* and *Nitrospira*), four fungal taxa (*Bionectria, Chaetomium, Exophiala* and *Fusarium*) and two protists (*Eocercomonas* and *Rhogostoma*) were common between the two studies (Fig. 2), demonstrating that the determination of the core microbiome is challenging and that the most appropriate method to do this has not yet been identified. For example, networks have been used to identify keystones species of wheat [35], [69] and DESeq2 has been used as a tool to identify both the core and differentially abundant taxa within treatments [27], [36], [38], [42], [56], [72] (Fig. 2). No genus was found to be common among all these different studies. *Sphingomonas* was detected in 80% of the studies; *Bradyrhizobium* in 70%; *Massilia* and *Pseudomonas* in 60%; and *Arthrobacter, Chitinophaga, Flavobacterium, Mucilaginibacter, Pantoea, Pedobacter* and *Variovorax* in 50% of the studies. It is important to highlight that the list of genera observed in Fig. 2 is not exhaustive, and the absence of other genera does not mean they are not present in those samples. It means that using the methods and tools available, these genera were found to be differentially abundant or were found to be keystone taxa when the different factors were considered.

With the definition of the core microbiome, it is possible to identify permanent community members as opposed to stochastic contributors for a given niche [109]. The recovery of representatives of such genera using culture-dependent methods and subsequent testing of their functional abilities both *in vitro* and *in planta* could be a strategy for the development of new inoculants. It follows that due to the phenomenon of functional redundancy, a true core microbiome based on taxonomy does not exist and that the core microbiome is a functional phenomenon, based on the presence of key genes which are not assessed in a taxonomical approach.

### 3.1 Putative PGPR associated with wheat

Microbial communities in soil influence plant health, growth, and resource use efficiency, especially the subset that is selected by plants to form the root microbiome [110], [111]. Bioprospecting microbes with plant growth-promoting (PGP) traits to increase productivity is a promising alternative to agrochemical application [112]. Plant growth-promoting rhizobacteria (PGPR) can influence plants through direct and indirect mechanisms [113]. Goswami et al. [114] define direct PGP activity as any mechanism that directly enhances plant growth. Examples include phytohormone production such as abscisic acid, indole 3-acetic acid (IAA), gibberellin, cytokinin, and ethylene; nutrient (nitrogen, phosphorus, potassium and zinc) solubilization; nitrogen fixation, and siderophore production. Indirect mechanisms protect plants from infections and abiotic environmental stresses via the production of enzymes (cellulase, chitinase, protease), volatiles (ammonia, hydrogen cyanide), bioactive secondary metabolites, and osmolytes [115], [116].

There is great potential for isolated bacteria to be used in improving wheat growth and many genera have been described in the literature as being capable of promoting plant growth. We searched the literature for specific PGP properties in bacterial genera commonly associated with wheat (Fig. 2), with search results being displayed in Table 2.

**Table 2:** Bacterial genera frequently associated with wheat which have been found to demonstrate putative PGP functions.

Phylum (Class)*	Genus	PGP	
		Function	Source
Actinobacteriota	*Aeromicrobium*	Phosphate solubilization, IAA and NH_3_ production [117]	Cold desert [117]
	*Arthrobacter*	Phosphate solubilization, IAA, siderophore, NH_3_ and GA production [117]; phosphate and zinc solubilization, IAA, siderophore, NH_3_ and ACC production, nitrogen fixation and biocontrol of *Fusarium graminearum*, *Rhizoctonia solani* and *Macrophomina phaseolina*[118]; putative N_2_ fixation [119]	Cold desert [117]; wheat [118]; wheat rhizosphere [119]
	*Streptomyces*	Phosphate solubilization and siderophore, IAA and extracellular enzymes (chitinase, alkaline protease, phytase, cellulase) production [120]	Wheat rhizosphere [120]

**Bacteroidota**	*Chitinophaga*	Putative N_2_ fixation [119]	Wheat rhizosphere and endosphere [119]
	*Chryseobacterium*	Phosphate, zinc and potassium solubilization, IAA, ACC, siderophore, NH_3_, protease, cellulase and lipase production [121]	Wheat rhizosphere [121]
	*Dyadobacter*	Phosphate solubilization [122]; nitrogen fixation [123]	Wheat rhizosphere [122]; bulk soil [123]
	*Flavobacterium*	Phosphate and zinc solubilization, IAA, siderophore, HCN, NH_3_ and ACC production [118]; phosphate and zinc solubilization, IAA, ACC, siderophore and NH_3_ production [121]	Wheat [118]; wheat rhizosphere [121]
	*Mucilaginibacter*	EPS production [124]; IAA production [125]	Rhizoplane of *Angelica sinensis*[124]; endosphere of *Tylosema esculentum*[125]
	*Segetibacter*	Not available	Bulk soil from ginseng field [126]

**Firmicutes**	*Bacillus*	Phosphate, potassium and zinc solubilization, IAA, siderophore, GA, HCN, NH_3_ and ACC production, nitrogen fixation and biocontrol of *Fusarium graminearum*, *Rhizoctonia solani* and *Macrophomina phaseolina phaseolina*[118]; putative N_2_ fixation [119]; zinc solubilization, IAA, ACC, NH_3_, protease, and cellulase production [121]	Wheat [118]; wheat rhizosphere and endosphere [119]; wheat rhizosphere [121]
	*Paenibacillus*	Phosphate solubilization and NH_3_ and IAA production [127]	Wheat rhizosphere [127]

**Gemmatimonadota**	*Gemmatimonas*	Not available	Anaerobic–aerobic sequential batch wastewater treatment reactor [128]

**Myxococcota**	*Haliangium*	Antifungal production [129]	Seaweed [129]

**Proteobacteria (Alphaproteobacteria)**	*Bradyrhizobium*	IAA production, protease and cellulolytic activity [130]	Seed endosphere of soybean [130]
	*Brevundimonas*	IAA, siderophore, GA and NH_3_ production and biocontrol of *Fusarium graminearum*, *Rhizoctonia solani* and *Macrophomina phaseolina*[118]; NH3 and IAA production and phosphate solubilization [127]	Wheat [118]; wheat rhizosphere [127]
	*Caulobacter*	IAA production and ARA [131]; plant growth promotion [132]	Rice endosphere [131]; maize endosphere [132]
	*Devosia*	Nitrogen fixation [133]; biocontrol of *Fusarium graminearum*[134]	Root nodules of *Neptunia natans*[133]; wheat field soil [134]
	*Rhizobium*	IAA, HCN and NH_3_ production and heavy metal tolerance [135]	Wheat rhizosphere [135]
	*Sphingomonas*	Nitrogen fixation, phosphate solubilization, siderophore, IAA, and ACC deaminase production [136]	Maize endosphere [136]

**Proteobacteria (Gammaproteobacteria)**	*Burkholderia*	ACC deaminase and IAA production [137]	Wheat rhizosphere [137]
	*Massilia*	IAA, siderophore and protease production [125]	Endosphere of marama bean (*Tylosema esculentum*) [125]
	*Pantoea*	Zinc solubilization, IAA, siderophore, GA, HCN, NH_3_ and ACC production, nitrogen fixation and biocontrol of *Fusarium graminearum*, *Rhizoctonia solani* and *Macrophomina phaseolina*[118]	Wheat [118]
	*Pedobacter*	IAA production [138]	Fertilized soil [138]
	*Pseudomonas*	Phosphate and zinc solubilization, IAA, siderophore, GA, HCN, NH_3_ and ACC production, nitrogen fixation and biocontrol of *Fusarium graminearum*, *Rhizoctonia solani* and *Macrophomina phaseolina*[118]); phosphate, zinc and potassium solubilization, IAA, ACC, siderophore, NH_3_, EPS, protease, and lipase production [121]; NH3, HCN and IAA production and antifungal activity against *Macrophomina phaseolina*[127]	Wheat [118]; wheat rhizosphere [121]
	*Rhodanobacter*	IAA production, phosphate solubilization and antifungal activity against *Cylindrocarpon destructans* and *Fusarium solani*[139]	Ginseng rhizosphere [139]
	*Stenotrophomonas*	Phosphate and potassium solubilization, IAA, siderophore, GA, HCN, NH_3_ and ACC production, nitrogen fixation and biocontrol of *Fusarium graminearum*, *Rhizoctonia solani* and *Macrophomina phaseolina*[118]; zinc solubilization, IAA, ACC, siderophore and NH_3_ production [121]	Wheat [118]; wheat rhizosphere [121]
	*Variovorax*	Inorganic phosphate solubilization [140]; ACC deaminase, siderophore and IAA production and cadmium tolerance [141]	Bulk soil [140]; indian mustard (*Brassica juncea*) rhizosphere [141]

ACC – 1-aminocyclopropane-1-carboxylate; ARA - acetylene reduction activity; EPS – exopolysaccharide; GA – gibberelic acid; HCN – hydrogen cyanide; IAA – indole 3-acetic acid; NH_3_ – ammonia.

*Taxonomy classification according to the Genome Taxonomy Database (GTDB) [142].

It should be noted that not all PGP functions described in Table 2 were observed in wheat. However, the fact that these bacteria are commonly associated with wheat does suggest that they could perform PGP activities in this crop. However, an important point is that the taxonomic affiliation of a bacterial isolate does not necessarily mean that it will perform a particular function. For example, *Rhizobium* spp. isolated in the UK are not able to fix nitrogen because they lack genes associated with this biosynthetic pathway [143].

Another consideration for the use of PGP bacteria is their ease of culturability. Although Table 2 was based on PGP function in bacterial cultures, it should be noted that some genera are more difficult to culture than others. For example, *Segetibacter koreensis* has been isolated from soil from a ginseng field in South Korea [126]. Additionally, a *Gemmatimonas* strain was obtained from an anaerobic–aerobic sequential batch wastewater treatment reactor [128]. Although widely spread in different environments, not many members of *Gemmatimonas* have been successfully cultivated [144] (Chee-Sanford et al. 2019). The genus *Haliangium* comprises myxobacteria with potential to produce bioactive secondary metabolites however, they are also hard to culture [145]. This highlights the need for improving and developing novel cultivation methods [146].

## 4 Gaps - how far are we from achieving a microbiome-facilitated sustainable agriculture?

The improvement of sequencing technologies has facilitated researchers to assess microbial communities in unprecedented detail. However, the deployment of microbes into agriculture has many challenges [147], [148]. Some of these are related to the formulation of microbes, their susceptibility to stresses, and their ability to colonize different niches in the face of competition from indigenous microbes, as well as the in-field expression of the desirable function and warranty of their safety to native organisms and the environment. Sessitch et al. [148] highlighted that one of the main difficulties in moving towards field application is that trial screenings are performed in a way that does not mimic real conditions. Hu et al. [62] used a portable DNA sequencer to detect plant pathogens and analyze the microbiome of infected wheat. They suggest that a combination of on-site and centralized sequencing approaches would, in the future, revolutionize the management of agricultural biosecurity and reduce crop losses.

Other challenges, which will be explored in detail, in addition to improving the culturability of potential microbes, include combining different “omics” approaches towards a better understanding of the potential of microbiomes, the development of synthetic communities, and the identification of a global wheat core microbiome. These are important gaps that need to be addressed before microbiomes can be successfully and fully implemented in agriculture.

### 4.1 Multidisciplinary approach

It is well known that a great variety of microbes are associated with crop plants. Conventionally, this interaction has been studied with a culture-based approach, often with the inoculation of a single microbial species. A better understanding of patterns of microbiome assemblage and manipulation is of fundamental importance for microbiome utilization. However, as these sequencing approaches are correlative, there remains a dependency on culture-based techniques for the successful application of microbes to the environment. In addition, it is desirable to obtain a genome sequence of a microbe of interest, and this is best achieved from a pure culture of a given microbe, as opposed to the computational assembly from metagenomes, where it can be difficult to accurately associate core and accessory genetic elements to a particular genome. Until recently only around 1% of bulk soil microbes and up to 10% of root-associated microbes were amenable to culture. However, dilution-to-extinction [149], the development of ichip [150], co-culturing, and other methods [151], have improved culture-based recovery of the soil and root-associated microbiome dramatically, thus the “1% culturability paradigm” needs to be revisited [152] and this is likely to facilitate the isolation of new species with important functions to benefit the plant host. As suggested by Schlaeppi and Bulgarelli [153], it might be useful to apply a combination of both culture-independent methods with culture-dependent methods to enable the development of inoculants towards a more reliable sustainable agriculture intensification. 16S rRNA gene and ITS amplicon analysis, shotgun metagenomics or metatranscriptomics could be used to detect changes in microbial communities, whereas cultivation techniques would be used to characterize the physiological properties of microorganisms. Although cultivation-based techniques present some limitations [36], Gutleben et al. [154] suggest they are currently the most reliable way to validate ecological hypotheses. The combination of different methods has important implications for the field of microbial ecology [155] and it has been demonstrated [156]. The taxa identified in the previous section could be used in the future for a targeted approach using culture-dependent methods coupled with culture-independent methods to enable the characterization and isolation of promising microorganisms for the development of synthetic communities (SynComs) will be further discussed in Section 4.3.

Additionally, the functional screening of microbial isolates using traditional culture-based methods focusing on the functions of single isolates are generally not high-throughput and have a low resolution. To overcome this, next-generation physiology approaches on microbial ecology studies to study the functions of microorganisms as communities in their native environment could be applied [157]. In addition, the culturability of “unculturable” microbes must be improved either by developing new cultivation strategies or by refining the existing ones.

**Fig. 3:**
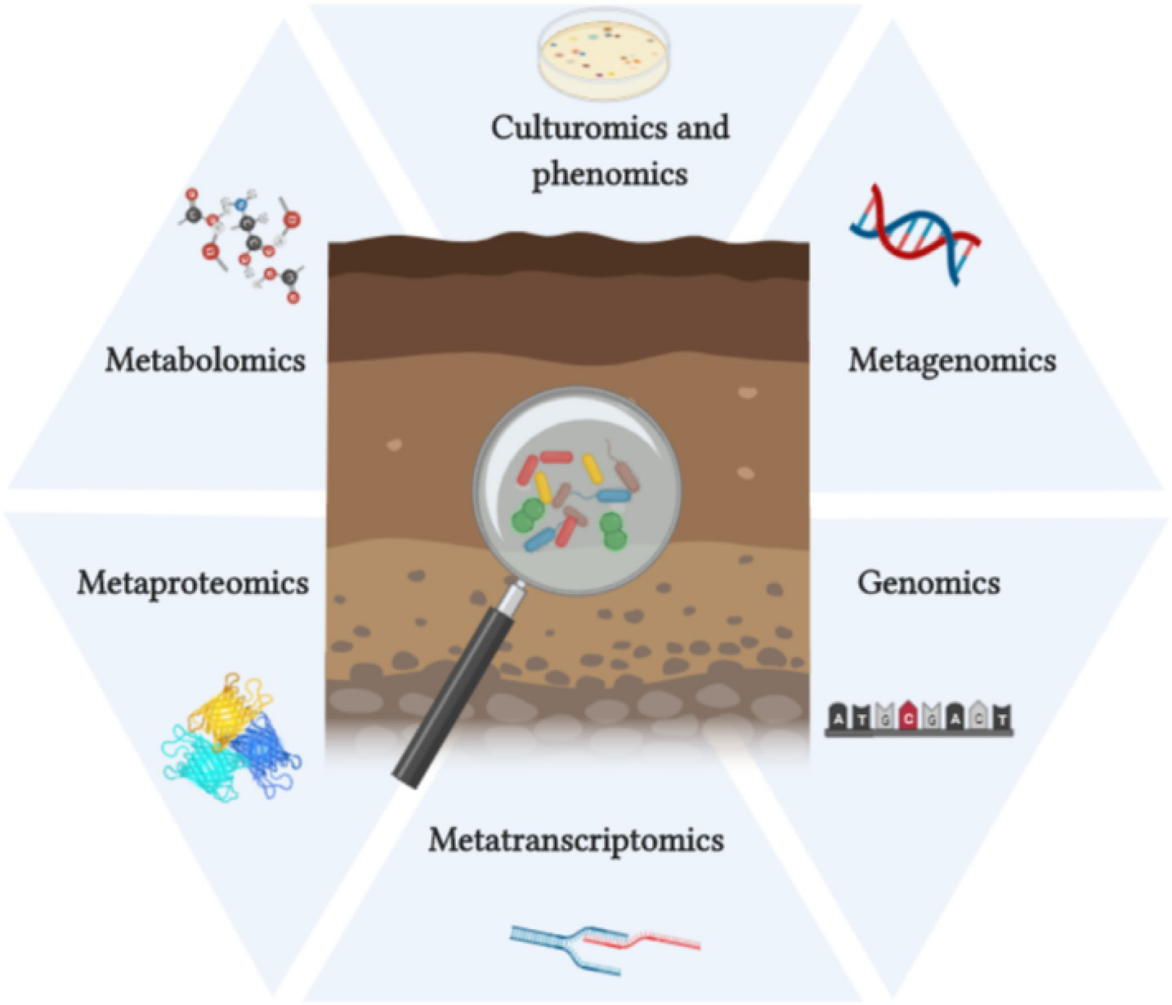
Proposed multidisciplinary framework for the successful use of microbiome in agriculture. Factors affecting the microbiome must be assessed through metagenomics (amplicon and shotgun), resulting in the description of the structure and diversity of microbial communities. Active microbial communities and genes should be assessed via metatranscriptomics. Additionally, culture-based methods should be used to recover isolates of interest (culturomics) and their functional and metabolic abilities evaluated by phenomics. Genomics can be used for targeting single cells or genes of interest using classical genetic approaches. And the effect of microbial inoculants on plant performance can be verified through metaproteomics (host-level) or metabolomics in the rhizosphere. Created with BioRender.com.

Researchers should combine ecological studies, and database information on the physiology and biochemistry of target isolates to efficiently uncover phylogenetically and functionally new strains [158]. Data from amplicon and metagenomics sequencing are quite descriptive and should be combined with other “omics” data such as metatranscriptomics and metabolomics to obtain a holistic description of factors affecting the wheat microbiome. Additionally, as already discussed, culturomics [158] should be used to isolate potential microbial candidates, alongside with phenomics data [159], where the metabolic and functional features of microbes are evaluated. Once isolates are obtained, single-cell genomics can be used for targeting genes of interest for classical genetics approaches, such as mutagenesis, deletion and complementation to prove the functional ability of the selected microbes. Finally, the effect of microbial inoculants on plants’ performance can be verified through metaproteomics (host-level) or metabolomics in the rhizosphere (Fig. 3). Understanding how plant’s metabolites select different microbes is a field of research that has been receiving more attention. By identifying which root metabolites are responsible for the proliferation of specific microbes, root exudates can be purified or synthesized and used to increase the host’s ability to recruit a beneficial microbiome [160]. However, several bottlenecks have been identified by Reuben et al. [161], such as the cost and technical constraints to detect different metabolites, the absence of a well-curated database and chemoinformatics tools to enable analysis and interpretation of collected data. In the future, if limitations related to techniques, analyses, and integration with other mentioned “omics” sciences are overcome, incorporating metabolomics studies into microbiome studies would enable engineering of the native soil microbiome for increased plant growth and performance under bespoke conditions.

### 4.2 Identification of the real core microbiome

Describing the core microbiome of a healthy host would facilitate the design of synthetic microbial communities that are more likely to establish under specific conditions. However, translating the findings towards the development of new inoculants will require a further assessment of their culturability and functionality under desired conditions both in glasshouse and field trials. Additionally, future research should focus on a benchmarking of all publicly available wheat root microbiome datasets. This study would provide insights into the degree of microbial functional redundancy in these systems and whether a taxonomically based global core wheat root microbiome exists, regardless of anthropogenic, edaphic, environmental and host-related factors.

### 4.3 Synthetic communities (SynComs) and the development of inoculants

The studies conducted on the wheat microbiome have highlighted which microbial communities are commonly associated with wheat and the factors responsible for the assembly of these communities. They might also offer hints to the identification of core representatives with possible plant growth-promoting traits, which could be used as inoculants or combined with other microbes into SynComs, which are artificially created by co-culturing two or more microbial strains in a specific medium [162]. Normally, they are designed for hypothesis testing and the selection of the members of these communities can be based on phylogeny, classification, networks or specific functions [163], always taking into account the ecological interactions among the different taxa [162]. Microbial inoculants combine a native population of microbes with several kinds of compounds, such as plant hormones and growth regulators which are produced and released during fermentation [164]. Ahemad and Khan [165] state that the exploitation of bacteria with multiple plant growth-promoting traits is beneficial, however, finding one bacterial strain with all desirable characteristics with the ability to colonize a variety of plant hosts and soil types is unlikely [166], making the use of mixtures of microbes, also known as synthetic communities a good alternative. García-Jiménez et al. [167] point out there are important considerations when designing SynComs such as how the communities will be structured to ensure stability and the desired output. It is therefore essential to understand the compatibility among the different members of a given synthetic community so that when co-inoculated they benefit the host, are not antagonistic toward one another, and are resilient when challenged with biotic and/or abiotic stresses. Although several studies have demonstrated the potential of different microbes to improve plant performance under different conditions, others have shown microbial inoculants to give poor results. As such their successful deployment requires further methodological, technical, and theoretical advances before they can be considered as a reliable alternative to agrochemicals [160].

## 5 Summary and outlook

Advances in the understanding of structure, diversity and functions of microbial communities associated with wheat and accompanying factors have been achieved in the last decades. We foresee great potential of microbiome manipulation for biostimulation of beneficial members of the indigenous microbiome to boost host performance under abiotic and biotic stresses. Identifying core microbiome function and the microbial genera responsible for these functions would reveal microbial targets for *in situ* manipulation. Alternatively, another approach would be the bioinoculation, addition of PGPR as microbial formulations (synthetic communities). However it is clear that a better understanding of bespoke conditions for successful establishment of inoculants is still required, in concert with the rationalized use of agrochemicals.

## Funding

This research was funded by OCP S.A under the UM6P Rothamsted Cranfield Program, the Natural Environment Research Council (NERC) and the Biotechnology and Biological Sciences Research Council (BBSRC) under research program NE/N018125/1 LTS-M ASSIST – Achieving Sustainable Agricultural Systems www.assist.ceh.ac.uk. We also thank Bilateral BBSRC-Embrapa grant on “Exploitation of the wheat rhizosphere microbiome for sustainable wheat production” (BB/N016246/1); “Optimization of nutrients in soil-plant systems: How can we control nitrogen cycling in soil?” (BBS/E/C/00005196) and “S2N – Soil to nutrition – Work package 1 – Optimizing nutrient flows and pools in the soil–plant-biota system” (BBS/E/C/000I0310).

## Declaration of Competing Interest

The authors declare that they have no known competing financial interests or personal relationships that could have appeared to influence the work reported in this paper.

## References

[1] Bell G.D.H., Lupton F.G.H. (1987). The history of wheat cultivation. Wheat breeding.

[2] Laino P., Limonta M., Gerna D., Vaccino P. (2015). Morpho-physiological and qualitative traits of a bread wheat collection spanning a century of breeding in Italy. Biodivers Data J.

[3] Taiz L. (2013). Agriculture, plant physiology, and human population growth: past, present, and future. Theor Exp Plant Phys.

[4] United Nations, Department of Economic and Social Affairs, Population Division (2019). World Population Prospects 2019: Highlights (ST/ESA/SER.A/423). Available at: https://population.un.org/wpp/Publications/Files/WPP2019_Highlights.pdf. Accessed 2020 September 28.

[5] OECD/FAO (2017) OECD-FAO Agricultural Outlook 2017-2026. OECD Publishing, Paris. Available at: 10.1787/agr_outlook-2017-en. Accessed 2020 September 28.

[6] Alexandratos N, Bruinsma J (2012) World agriculture towards 2030/2050: the 2012 revision. ESA Working paper No. 12-03. Rome, FAO. Available at: http://www.fao.org/3/ap106e/ap106e.pdf. Accessed 2020 September 28.

[7] Davis K.F., Gephart J.A., Emery K.A., Leach A.M., Galloway J.N. (2016). Meeting future food demand with current agricultural resources. Global Environ Chang.

[8] Dubey P.K., Singh G.S., Abhilash P.C. (2020). Adaptive agricultural practices – building resilience in a changing climate. Springer briefs in environmental science.

[9] Misra M., Sachan A., Sachan S.G., Yadav A.N., Singh J., Rastegari A.A., Yadav N. (2020). Current aspects and applications of biofertilizers for sustainable agriculture. Plant Microbiomes for Sustainable Agriculture, Sustainable Development and Biodiversity.

[10] Ruinen J. (1956). Occurrence of *Beijerinckia* species in the phyllosphere. Nature.

[11] Compant S., Clément C., Sessitsch A. (2010). Plant growth-promoting bacteria in the rhizo- and endosphere of plantsÇ their role, colonization, mechanisms involved and prospects for utilization. Soil Biol Biochem.

[12] Hiltner L. (1904). Über neuere Erfahrungen und Probleme auf dem Gebiete der Bodenbakteriologie unter besonderer Berücksichtigung der Gründüngung und Brache. Arb DLG.

[13] Clark F.E. (1949). Soil microorganisms and plant roots. Adv Agron.

[14] Hallmann J., Quadt-Hallmann A., Mahaffee W., Kloepper J. (1997). Bacterial endophytes in agricultural crops. Can J Microbiol.

[15] Perotti R. (1926). On the limits of biological inquiry on soil science. Proc Int Soc Soil Sci.

[16] Nelson E.B. (2004). Microbial dynamics and interactions in the spermosphere. Annu Rev Phytopathol.

[17] Verona O. (1958). La spermosphere. Ann Inst Pasteur.

[18] Karlsson I., Friberg H., Steinberg C., Persson P. (2014). Fungicide effects on fungal community composition in the wheat phyllosphere. PLoS One.

[19] Knorr K., Jørgensen L.N., Nicolaisen M. (2019). Fungicides have complex effects on the wheat phyllosphere mycobiome. PLoS One.

[20] Schlatter D.C., Yin C., Hulbert S., Burke I., Paulitz T. (2017). Impacts of repeated glyphosate use on wheat-associated bacteria are small and depend on glyphosate use history. Appl Environ Microbiol.

[21] Li Y., An J., Dang Z., Lv H., Pan W. (2018). Treating wheat seeds with neonicotinoid insecticides does not harm the rhizosphere microbial community. PLoS One.

[22] Solanki M.K., Abdelfattah A., Britzi M., Zakin V., Wisniewski M. (2019). Shifts in the composition of the microbiota of stored wheat grains in response to fumigation. Front Microbiol.

[23] Qi Y., Ossowicki A., Yang X., Lwanga E.H., Dini-Andreote F. (2020). Effects of plastic mulch film residues on wheat rhizosphere and soil properties. J Hazard Mater.

[24] Amadou A., Song A., Tang Z.-X., Li Y., Wang E.-Z. (2020). The effects of organic and mineral fertilization on soil enzyme activities and bacterial community in the below- and above-ground parts of wheat. Agronomy.

[25] Chen S., Waghmode T.R., Sun R., Kuramae E.E., Hu C. (2019). Root-associated microbiomes of wheat under the combined effect of plant development and nitrogen fertilization. Microbiome.

[26] Illescas M., Rubio M.B., Hernández-Ruiz V., Morán-Diez M.E., Martínez de Alba A.E. (2020). Effect of inorganic N top dressing and *Trichoderma harzianum* seed-inoculation on crop yield and the shaping of root microbial communities of wheat plants cultivated under high basal N fertilization. Front Plant Sci.

[27] Kavamura V.N., Hayat R., Clark I.M., Rossmann M., Mendes R. (2018). Inorganic nitrogen application affects both taxonomical and predicted functional structure of wheat rhizosphere bacterial communities. Front Microbiol.

[28] Liu W., Ling N., Guo J., Ruan Y., Zhu C. (2020). Legacy effects of 8-year nitrogen inputs on bacterial assemblage in wheat rhizosphere. Biol Fertil Soils.

[29] Pagé A.P., Tremblay J., Masson L., Greer C.W. (2019). Nitrogen- and phosphorus-starved *Triticum aestivum* show distinct belowground microbiome profiles. PLoS One.

[30] Robinson R.J., Fraaije B.A., Clark I.M., Jackson R.W., Hirsch P.R. (2016). Endophytic bacterial community composition is determined by plant tissue type, developmental stage and soil nutrient availability. Plant Soil.

[31] Schmalenberger A., Hodge S., Hawkesford M.J., Kertesz M.A. (2009). Sulfonate desulfurization in *Rhodococcus* from wheat rhizosphere communities. FEMS Microbiol Ecol.

[32] Simonin M., Dasilva C., Terzi V., Ngonkeu E.L.M., Diouf D. (2020). Influence of plant genotype and soil on the wheat rhizosphere microbiome: evidences for a core microbiome across eight African and European soils. FEMS Microbiol Ecol.

[33] Yergeau É., Quiza L., Tremblay J. (2020). Microbial indicators are better predictors of wheat yield and quality than N fertilization. FEMS Microbiol Ecol.

[34] Araujo R., Dunlap C., Barnett S., Franco C.M.M. (2019). Decoding wheat endosphere rhizosphere microbiomes in *Rhizoctonia solani*-infested soils challenged by *Streptomyces* biocontrol agents. Front Plant Sci.

[35] Araujo R., Dunlap C., Barnett S., Franco C.M.M. (2020). Analogous wheat root rhizosphere microbial successions in field and greenhouse trials in the presence of biocontrol agents *Paenibacillus peoriae* SP9 and *Streptomyces fulvissimus* FU14. Mol Plant Pathol.

[36] Kavamura V.N., Robinson R.J., Hayat R., Clark I.M., Hughes D. (2019). Land management and microbial seed load effect on rhizosphere and endosphere bacterial community assembly in wheat. Front Microbiol.

[37] Rossmann M., Pérez-Jaramillo J.E., Kavamura V.N., Chiaramonte J.B., Dumack K. (2020). Multitrophic interactions in the rhizosphere microbiome of wheat: from bacteria and fungi to protists. FEMS Microbiol Ecol.

[38] a Schlatter DC, Yin C, Hulbert S, Paulitz T, Core rhizosphere microbiomes of dryland wheat are influenced by location and land use history Appl Environ Microbiol 86 5 2020 e02135 19 DOI:10.1128/AEM.02135-19. 10.1128/AEM.02135-19 PMC7028972 31862727

[39] Gdanetz K., Trail F. (2017). The wheat microbiome under four management strategies, and potential for endophytes in disease protection. Phytobiomes J.

[40] Hartman K., van der Heijden M.G.A., Wittwer R.A., Banerjee S., Walser J.-C. (2018). Cropping practices manipulate abundance patterns of root and soil microbiome members paving the way to smart farming. Microbiome.

[41] Ishaq S.I., Seipel T., Yeoman C.J., Menalled F.D. (2020). Soil bacterial communities of wheat vary across the growing season and among dryland farming systems. Geoderma.

[42] Mavrodi D.V., Mavrodi O.V., Elbourne L.D.H., Tetu S., Bonsall R.F. (2018). Long-term irrigation affects the dynamics and activity of the wheat rhizosphere microbiome. Front Plant Sci.

[43] Donn S., Kirkegaard J.A., Perera G., Richardson A.E., Watt M. (2014). Evolution of bacterial communities in the wheat crop rhizosphere. Environ Microbiol.

[44] Lupwayi N.Z., Rice W.A., Clayton G.W. (1998). Soil microbial diversity and community structure under wheat as influenced by tillage and crop rotation. Soil Biol Biochem.

[45] Mayer Z., Sasvári Z., Szentpéteri V., Rétháti B.P., Vajna B. (2019). Effect of long-term cropping systems on the diversity of the soil bacterial communities. Agronomy.

[46] Wen X.-Y., Dubinsky E., Wu Y., Yu R., Chen F. (2016). Wheat, maize and sunflower cropping systems selectively influence bacteria community structure and diversity in their and succeeding crop’s rhizosphere. J Integr Agric.

[47] Xiong C., Zhu Y.-G., Wang J.-T., Singh B., Han L.-L. (2020). Host selection shapes crop microbiome assembly and network complexity. New Phytol.

[48] Yin C., Jones K.L., Peterson D.E., Garret K.A., Hulbert S.H. (2010). Members of soil bacterial communities sensitive to tillage and crop rotation. Soil Biol Biochem.

[49] Yin C., Mueth N., Hulbert S., Schlatter D., Paulitz T.C. (2017). Bacterial communities on wheat grown under long-term conventional tillage and no-till in the Pacific Northwest of the United States. Phytobiomes J.

[50] b Schlatter DC, Kahl K, Carlson B, Huggins DR, Paulitz T, Soil acidification modifies soil depth-microbiome relationships in a no-till wheat cropping system Soil Biol Biochem 107939 2020 DOI:10.1016/j.soilbio.2020.107939.

[51] Uksa M., Buegger F., Gschwendtner S., Lueders T., Kublik S. (2017). Bacteria utilizing plant-derived carbon in the rhizosphere of *Triticum aestivum* change in different depths of an arable soil. Environ Microbiol Rep.

[52] Azarbad H., Tremblay J., Giard-Laliberté, Bainard L.D., Yergeau E. (2020). Four decades of soil water stress history together with host genotype constrain the response of the wheat microbiome to soil moisture. FEMS Microbiol Ecol.

[53] Fan K., Cardona C., Li Y., Shi Y., Xiang X. (2017). Rhizosphere-associated bacterial network structure and spatial distribution differ significantly from bulk soil in wheat crop fields. Soil Biol Biochem.

[54] Fan K., Weisenhorn P., Gilbert J.A., Chu H. (2018). Wheat rhizosphere harbors a less complex and more stable microbial co-occurrence pattern than bulk soil. Soil Biol Biochem.

[55] Wolińska A., Kuźniar A., Gałązka A. (2020). Biodiversity in the rhizosphere of selected winter wheat (*Triticum aestivum* L.) cultivars – genetic and catabolic fingerprinting. Agronomy.

[56] Schlatter D.C., Hansen J.C., Schillinger W.F., Sullivan T.S., Paulitz T.C. (2019). Common and unique rhizosphere microbial communities of wheat and canola in a semiarid Mediterranean environment. Appl Soil Ecol.

[57] Jochum M.D., McWilliams K.L., Pierson E.A., Jo Y.-K. (2019). Host-mediated microbiome engineering (HMME) of drought tolerance in the wheat rhizosphere. PLoS One.

[58] Latz M.A.C., Kerrn M.H., Sørensen H., Collinge D.B., Jensen B. (2021). Succession of the fungal endophytic microbiome of wheat is dependent on tissue-specific interactions between host genotype and environment. Sci Total Environ.

[59] Naylor D., DeGraaf S., Purdom E., Coleman-Derr D. (2017). Drought and host selection influence bacterial community dynamics in the grass root microbiome. ISME J.

[60] Stromberger M.E., Abduelafez I., Byrne P., Canela M.M., Elamari A.A. (2017). Genotype-specific enrichment of 1-aminocyclopropane-1-carboxylic acid deaminase-positive bacteria in winter wheat rhizospheres. Soil Biol Biochem.

[61] Hayden H.L., Savin K.W., Wadeson J., Gupta V.V.S.R., Mele P.M. (2018). Comparative metatranscriptomics of wheat rhizosphere microbiomes in disease suppressive and non-suppressive soils for *Rhizoctonia solani* AG8. Front Microbiol.

[62] Hu Y., Green G.S., Milgate A.W., Stone E.A., Rathjen J.P. (2019). Pathogen detection and microbiome analysis of infected wheat using a portable DNA sequencer. Phytobiomes J.

[63] Kerdraon L., Barret M., Laval V., Suffert F. (2019). Differential dynamics of microbial community networks help identify microorganisms interacting with residue-borne pathogens: the case of *Zymoseptoria tritici* in wheat. Microbiome.

[64] Rojas E.C., Sapkota R., Jensen B., Jørgensen H.J.L., Henriksson T. (2020). Fusarium head blight modifies fungal endophytic communities during infection of wheat spikes. Microb Ecol.

[65] Seybold H., Demetrowitsch T.J., Hassani M.A., Szymczak S., Reim E. (2020). A fungal pathogen induces systemic susceptibility and systemic shifts in wheat metabolome and microbiome composition. Nat Commun.

[66] Yin C., Hulbert S.H., Schroeder K.L., Mavrodi O.A. (2013). Role of bacterial communities in the natural suppression of *Rhizoctonia solani* bare patch disease of wheat (*Triticum aestivum* L.). Appl Environ Microbiol.

[67] Cordero J., Freitas J.R., Germida J.J. (2020). Bacterial microbiome associated with the rhizosphere and root interior of crops in Saskatchewan, Canada. Can J Microbiol.

[68] Latif S., Bibi S., Kouser R., Fatimah H., Farooq S. (2020). Characterization of bacterial community structure in rhizosphere of *Triticum aestivum* L. Genomics.

[69] Mahoney A.K., Yin C., Hulbert S.H. (2017). Community structure, species variation, and potential functions of rhizosphere-associated bacteria of different winter wheat (*Triticum aestivum*) cultivars. Front Plant Sci.

[70] Sapkota R., Jørgensen L.N., Nicolaisen M. (2017). Spatiotemporal variation and networks in the mycobiome of the wheat canopy. Front Plant Sci.

[71] Hassani M.A., Özkurt E., Franzenburg S., Stukenbrock E.H. (2020). Ecological assembly processes of the bacterial and fungal microbiota of wild and domesticated wheat species. Phytobiomes J.

[72] Kavamura V.N., Robinson R.J., Hughes D., Clark I., Rossmann M. (2020). Wheat dwarfing influences selection of the rhizosphere microbiome. Sci Rep.

[73] Kinnunen-Grubb M., Sapkota R., Vignola M., Nunes I.M., Nicolaisen M. (2020). Breeding selection imposed a differential selective pressure on the wheat root-associated microbiome. FEMS Microbiol Ecol.

[74] Sun X., Kosman E., Sharon A. (2020). Stem endophytic mycobiota in wild and domesticated wheat: structural differences and hidden resources for wheat improvement. J Fungi.

[75] Tkacz A., Pini F., Turner T.R., Bestion E., Simmonds J. (2020). Agricultural selection of wheat has been shaped by plant-microbe interactions. Front Microbiol.

[76] Valente J., Gerin F., Le Gouis J., Moënne-Loccoz Y., Prigent-Combaret C. (2019). Ancient wheat varieties have a higher ability to interact with plant growth-promoting rhizobacteria. Plant Cell Environ.

[77] Mauchline T.H., Chedom-Fotso D., Chandra G., Samuels T., Greenaway N. (2015). An analysis of *Pseudomonas* genomic diversity in take-all infected wheat fields reveals the lasting impact of wheat cultivars on the soil microbiota. Environ Microbiol.

[78] Zuo S., Li X., Ma Y., Yang S. (2014). Soil microbes are linked to the allelopathic potential of different wheat genotypes. Plant Soil.

[79] Huang Y., Kuang Z., Wang W., Cao L. (2016). Exploring potential bacterial and fungal biocontrol agents transmitted from seeds to sprouts of wheat. Biol Control.

[80] Kuźniar A., Włodarczyk K., Grządziel J., Goraj W., Gałązka A. (2020). Culture-independent analysis of an endophytic core microbiome in two species of wheat: *Triticum aestivum* L. (cv. ‘Hondia’) and the first report of microbiota in *Triticum spelta* L. (cv. ‘Rokosz’). Syst Appl Microbiol.

[81] Liu H., Carvalhais L.C., Schenk P.M., Dennis P.G. (2017). Effects of jasmonic acid signalling on the wheat microbiome differ between body sites. Sci Rep.

[82] Liu H., Carvalhais L.C., Schenk P.M., Dennis P.G. (2018). Activation of the salicylic acid signalling pathway in wheat had no significant short-term impact on the diversity of root-associated microbiomes. Pedobiologia.

[83] Ansari M.S., Moraiet M.A., Ahmad S., Malik A., Grohmann E., Akhtar R. (2014). Insecticides: impact on the environment and human health. Environmental deterioration and human health.

[84] Van Bruggen A.H.C., He M.M., Shin K., Mai V., Jeong K.C. (2018). Environmental and health effects of the herbicide glyphosate. Sci Total Environ.

[85] Malalgoda M., Ohm J.-B., Howatt K.A., Simsek S. (2020). Pre-harvest glyphosate application and effects on wheat starch chemistry: analysis from application to harvest. J Food Biochem.

[86] Köhl J., Booij K., Kolnaar R., Ravensberg W.J. (2019). Ecological arguments to reconsider data requirements regarding the environmental fate of microbial biocontrol agents in the registration procedure in the European Union. Biocontrol.

[87] Önder M, Ceyhan E, Kahraman A (2011) Effects of agricultural practices on environment. IPCBEE, 24. Available at: http://www.ipcbee.com/vol24/6-ICBEC2011-C00015.pdf. Accessed 2020 September 28.

[88] Shakoor A., Shahbaz M., Farooq T.H., Sahar N.E., Shahzad S.M. (2021). A global meta-analysis of greenhouse gases emission and crop yield under no-tillage as compared to conventional tillage. Sci Total Environ.

[89] Hirsch P.R., Jhurreea D., Williams J.K., Murray P.J., Scott T. (2017). Soil resilience and recovery: rapid community responses to management changes. Plant Soil.

[90] Chapelle E., Mendes R., Bakker P.A.H.M., Raaijmakers J.M. (2016). Fungal invasion of the rhizosphere microbiome. ISME J.

[91] Raaijmakers J.M., Mazzola M. (2016). Soil immune responses. Science.

[92] Lamoureux EV, Grandy SA, Langille MGI (2017) Moderate exercise has limited but distinguishable effects on the mouse microbiome. mSystems 2(4):1-14. 10.1128/mSystems.00006-17 PMC5566786 28845459

[93] Vandenkoornhuyse P., Quaiser A., Duhamel M., Le Van A., Dufresne A. (2015). The importance of the microbiome of the plant holobiont. New Phytol.

[94] Jones P., Garcia B.J., Furches A., Tuskan G.A., Jacobson D. (2019). Plant host-associated mechanisms for microbial selection. Front Plant Sci.

[95] Teixeira P.J.P.L., Colaianni N.R., Fitzpatrick C.R., Dangl J.L. (2019). Beyond pathogens: microbiota interactions with the plant immune system. Curr Opin Microbiol.

[96] Chu H, Gao G-F, Ma Y, Fan K, Delgado-Baquerizo M (2020) Soil microbial biogeography in a changing world: recent advances and future perspectives. mSystems 5:e00803-19. DOI:10.1128/mSystems.00803-19. 10.1128/mSystems.00803-19 PMC7174637 32317392

[97] Beckers B., De Beeck N.O., Weyens N., Boerjan W., Vangronsveld J. (2017). Structural variability and niche differentiation in the rhizosphere and endosphere bacterial microbiome of field-grown poplar trees. Microbiome.

[98] Hedden P. (2003). The genes of the green revolution. Trends Genet.

[99] Law C.N., Snape J.W., Worland A.J. (1978). The genetical relationship between height and yield in wheat. Heredity.

[100] Bertin C., Yang X.H., Weston L.A. (2003). The role of root exudates and allelochemicals in the rhizosphere. Plant Soil.

[101] Graaff M.A., Six J., Jastrow J.D., Schadt C.W., Wullschleger S.D. (2013). Variation in root architecture among switchgrass cultivars impacts root decomposition rates. Soil Biol Biochem.

[102] Shade A., Stopnisek N. (2019). Abundance-occupancy distributions to prioritize plant core microbiome membership. Curr Opin Microbiol.

[103] Toju H., Peay K.G., Yamamichi M., Narisawa K., Hiruma K. (2018). Core microbiomes for sustainable agroecosystems. Nat Plants.

[104] Lahti L, Shetty S, et al. (2017). Tools for microbiome analysis in R. Version 2.1.26. URL: http://microbiome.github.com/microbiome.

[105] Cernava T., Erlacher A., Soh J., Sensen C.W., Grube M. (2019). Enterobacteriaceae dominate the core microbiome and contribute to the resistome of arugula (*Eruca sativa* Mill.). Microbiome.

[106] Chopyk J., Akrami K., Bavly T., Shin J.H., Schwanemann L.K. (2020). Temporal variations in bacterial community diversity and composition throughout intensive care unit renovations. Microbiome.

[107] Douglas A.J., Hug L.A., Katzenback B.A. (2020). Composition of the North American wood frog (*Rana sylvatica*) bacterial skin microbiome and seasonal variation in community structure. Microb Ecol.

[108] Risely A. (2020). Applying the core microbiome to understand host-microbe systems. J Anim Ecol.

[109] Berg G., Rybakova D., Fischer D., Cernava T., Vergès M.C.C. (2020). Microbiome definition re-visited: old concepts and new challenges. Microbiome.

[110] Berendsen R.L., Pieterse C.M.J., Bakker P.A.H.M. (2012). The rhizosphere microbiome and plant health. Trends Plant Sci.

[111] Mendes R., Garbeva P., Raaijmakers J.M. (2013). The rhizosphere microbiome: significance of plant beneficial, plant pathogenic, and human pathogenic microorganisms. FEMS Microbiol Rev.

[112] Nagargade M., Tyagi V., Singh M.K., Meena V.S. (2018). Plant growth-promoting rhizobacteria: a biological approach toward the production of sustainable agriculture. Role of rhizospheric microbes in soil. Volume 1: Stress management and agriculture sustainability.

[113] Solano B.R., Barriuso J., Mañero F.J.G., Ahmad I., Pichtel J., Hayat S. (2008). Physiological and molecular mechanisms of plant growth promoting rhizobacteria (PGPR). Plant-bacteria interactions. Strategies and techniques to promote plant growth.

[114] Goswami D., Thakker J.N., Dhandhukia P.C. (2016). Portraying mechanics of plant growth promoting rhizobacteria (PGPR): a review. Cogent Food Agric.

[115] Saraf M., Rajkumar S., Saha T., Maheshwari D.K.K. (2011). Perspectives of PGPR in agri-ecosystems. Bacteria in agrobiology: crop systems.

[116] Tyc O., Song C., Dickschat J.S., Vos M., Garbeva P. (2017). The ecological role of volatile and soluble secondary metabolites produced by soil bacteria. Trends Microbiol.

[117] Yadav A.N., Sachan S.G., Verma P., Saxena A.K. (2014). Prospecting cold deserts of north western Himalayas for microbial diversity and plant growth promoting attributes. J Biosci Bioeng.

[118] Verma P., Yadav A.N., Khannam K.S., Panjiar N., Kumar S. (2015). Assessment of genetic diversity and plant growth promoting attributes of psychrotolerant bacteria allied with wheat (Triticum aestivum) from the northern hills zone of India. Ann Microbiol.

[119] Rilling J.I., Acuña J.J., Sadowsky M.J., Jorquera M.A. (2018). Putative nitrogen-fixing bacteria associated with the rhizosphere and root endosphere of wheat plants grown in an andisol from Southern Chile. Front Microbiol.

[120] Jog R., Nareshkumar G., Rajkumar S. (2012). Plant growth promoting potential and soil enzyme production of the most abundant *Streptomyces* spp. from wheat rhizosphere. J Appl Microbiol.

[121] Gontia-Mishra I., Sapre S., Kachare S., Tiwari S. (2017). Molecular diversity of 1-amynocyclopropane-1-carboxulate (ACC) deaminase producing PGPR from wheat (*Triticum aestivum* L.) rhizosphere. Plant Soil.

[122] Zhang J., Liu J., Meng L., Ma Z., Tang X. (2012). Isolation and characterization of plant growth-promoting rhizobacteria from wheat roots by wheat germ agglutinin labeled with fluorescein isothiocyanate. J Microbiol.

[123] Kumar S., Suyal D.C., Bhoriyal M., Goel R. (2018). Plant growth promoting potential of psychrotolerant *Dyadobacter* sp. for pulses and finger millet and impact of inoculation on soil chemical properties and diazotrophic abundance. J Plant Nutr.

[124] Han S.-I., Lee H.-J., Lee H.-R., Kim K.-K., Whang K.-S. (2012). *Mucilaginibacter polysacchareus* sp. nov., an exopolysaccharide-producing bacterial species isolated from the rhizoplane of the herb *Angelica sinensis*. Int J Syst Evol Microbiol.

[125] Chimwamurombe P.M., Grönemeyer J.L., Reinhold-Hurek B. (2016). Isolation and characterization of culturable seed-associated bacterial endophytes from gnotobiotically grown Marama bean seedlings. FEMS Microbiol Ecol.

[126] An D.-S., Lee H.-G., Im W.-T., Liu Q.-M., Lee S.-T. (2007). *Segetibacter koreensis* gen. nov., sp. nov., a novel member of the phylum Bacteroidetes, isolated from the soil of a ginseng field in South Korea. Int J Syst Evol Microbiol.

[127] Rana A., Saharan B., Joshi M., Prasanna R., Kumar K. (2011). Identification of multi-trait PGPR isolates and evaluating their potential as inoculants for wheat. Ann Microbiol.

[128] Zhang H., Sekiguchi Y., Hanada S., Hugenholtz P., Kim H. (2003). *Gemmatimonas aurantiaca* gen. nov., sp. nov., a Gram-negative, aerobic, polyphosphate-accumulating micro-organism, the first cultured representative of the new bacterial phylum Gemmatimonadetes phyl. nov. Evol Microbiol.

[129] Fudou R., Iizuka T., Yamanaka S. (2001). Haliangicin, a novel antifungal metabolite produced by a marine myxobacterium 1. Fermentation and biological characteristics. J Antibiot.

[130] Masciarelli O., Llanes A., Luna V. (2014). A new PGPR co-inoculated with *Bradyrhizobium japonicum* enhances soybean nodulation. Microbiol Res.

[131] Habibi S., Djedidi S., Prongjunthuek K., Mortuza M.F., Ohkama-Ohtsu N. (2014). Physiological and genetic characterization of rice nitrogen fixer PGPR isolated from rhizosphere soils of different crops. Plant Soil.

[132] Luo D., Langendries S., Mendez S.G., Ryck J., Liu D. (2019). Plant growth promotion driven by a novel *Caulobacter* strain. MPMI.

[133] Rivas R., Velázquez E., Willems A., Vizcaíno N., Subba-Rao N.S. (2002). A new species of *Devosia* that forms a unique nitrogen-fixing root-nodule symbiosis with the aquatic legume *Neptunia natans* (L.f.) Druce. Appl Environ Microbiol.

[134] Sato I., Ito M., Ishizawa M., Ikunaga Y., Sato Y. (2012). Thirteen novel deoxynivalenol-degrading bacteria are classified within two genera with distinct degradation mechanisms. FEMS Microbiol Lett.

[135] Singh Y., Lal N. (2016). Isolation and characterization of PGPR from wheat (*Triticum aestivum*) rhizosphere and their plant growth promoting traits *in vitro*. I J Biol.

[136] Correa-Galeote D., Bedmar E.J., Arone G.J. (2018). Maize endophytic bacterial diversity as affected by soil cultivation history. Front Microbiol.

[137] Shaharoona B., Jamro G.M., Zahir Z.A., Arshad M., Memon K.S. (2007). Effectiveness of various *Pseudomonas* spp. and *Burkholderia caryophylli* containing ACC-deaminase for improving growth and yield of wheat (*Triticum aestivum* L.). J Microbiol Biotechnol.

[138] Yuan C.-L., Mou C.-X., Wu W.-L., Guo Y.-B. (2011). Effect of different fertilization treatments on indole-3-acetic acid producing bacteria in soil. J Soils Sediments.

[139] Huo Y., Kang J.P., Ahn J.C., Kim Y.J., Piao C.H. (2020). Siderophore-producing rhizobacteria reduce heavy metal-induced oxidative stress in *Panax ginseng* Meyer. J Ginseng Res.

[140] Zheng B.-X., Ding K., Yang X.-R., Wadaan M.A.M., Hozzein W.N. (2019). Straw biochar increases the abundance of inorganic phosphate solubilizing bacterial community for better rape (*Brassica napus*) growth and phosphate uptake. Sci Total Environ.

[141] Belimov A.A., Hontzeas N., Safranova V.I., Demchinskaya S.V., Piluzza G. (2005). Cadmium-tolerant plant growth-promoting bacteria associated with the roots of Indian mustard (*Brassica juncea* L. Czern.). Soil Biol Biochem.

[142] Parks D.H., Chuvochina M., Waite D.W., Rinke C., Skarshewski A. (2018). A standardized bacterial taxonomy based on genome phylogeny substantially revises the tree of life. Nat Biotechnol.

[143] Jones F., Clark I., King R., Shaw L.J., Woodward M.J. (2016). Novel European free-living, non-diazotrophic *Bradyrhizobium* isolates from contrasting soils that lack nodulation and nitrogen fixation genes – a genome comparison. Sci Rep.

[144] Chee-Sanford J., Tian D., Sanford R. (2019). Consumption of N2O and other N-cycle intermedaites by *Gemmatimonas aurantiaca* strain T-27. Microbiology.

[145] Mohr K.I. (2018). Diversity of myxobacteria – we only see the tip of the iceberg. Microorganisms.

[146] Busby P.E., Soman C., Wagner M.R., Friesen M.L., Kremer J. (2017). Research priorities for harnessing plant microbiomes in sustainable agriculture. PLoS Biol.

[147] Parnell J.J., Berka R., Young H.A., Sturino J.M., Kang Y. (2016). From the lab to the farm: an industrial perspective of plant beneficial microorganisms. Front Plant Sci.

[148] Sessitch A., Pfaffenbichler N., Mitter B. (2019). Microbiome applications from lab to field: facing complexity. Trends Plant Sci.

[149] Song J., Oh H.M., Cho J.C. (2009). Improved culturability of SAR11 strains in dilution-to-extinction culturing from the East Sea, West Pacific Ocean. FEMS Microbiol Lett.

[150] Nichols D., Cahoon N., Trakhtenberg E.M., Pham L., Mehta A. (2010). Use of Ichip for high-throughput in situ cultivation of “uncultivable” microbial species. Appl Environ Microbiol.

[151] Stewart E.J. (2012). Growing unculturable bacteria. J Bacteriol.

[152] Martiny A.C. (2019). High proportions of bacteria are culturable across major biomes. ISME J.

[153] Schlaeppi K., Bulgarelli D. (2015). The plant microbiome at work. MPMI.

[154] Gutleben J., De Mares M.C., van Elsas J.D., Smidt H., Overmann J. (2018). The multi-omics promise in context: from sequence to microbial isolate. Crit Rev Microbiol.

[155] VanInsberghe D., Hartamnn M., Stewart G.R., Mohn W.M. (2013). Isolation of a substantial proportion of forest soil bacterial communities detected via pyrotag sequencing. Appl Environ Microbiol.

[156] Armanhi J.S.L., de Souza R.S.C., Damasceno N.B., de Araújo L.M., Imperial J. (2018). A community-based culture collection for targeting novel plant growth-promoting bacteria from the sugarcane microbiome. Front Plant Sci.

[157] Hatzenpichler R., Krukenberg V., Spietz R.L., Jay Z.J. (2020). Next-generation physiology approaches to study microbiome function at single cell level. Nat Rev Microbiol.

[158] Overmann J., Abt B., Sikorski J. (2017). Present and future of culturing bacteria. Annu Rev Microbiol.

[159] Alcin-Albiac M., Filannino P., Gobbetti M., Di Cagno R. (2020). Microbial high throughput phenomics: the potential of an irreplaceable omics. Comput Struct Biotechnol J.

[160] Qiu Z., Egidi E., Liu H., Kaur S., Singh B.K. (2019). New frontiers in agriculture productivity: optimised microbial inoculants and in situ microbiome engineering. Biotechnol Adv.

[161] Reuben S., Bhinu V.S., Swarup S., Karlovsky P. (2008). Rhizosphere metabolomics: methods and applications. Secondary metabolites in soil ecology.

[162] Großkopf T., Soyer O.S. (2014). Synthetic microbial communities. Curr Opin Microbiol.

[163] Vorholt J.A., Vogel C., Carlström C.I., Müller D.B. (2017). Establishing casuality: opportunities of synthetic communities for plant microbiome research. Cell Host Microbe.

[164] Cassán F., Perrig D., Sgroy V., Masciarelli O., Penna C. (2009). *Azospirillum brasilense* Az39 and *Bradyrhizobium japonicum* E109, inoculated singly or in combination, promote seed germination and early seedling growth in corn (*Zea mays* L.) and soybean (*Glycine max* L.). Eur J Soil Biol.

[165] Ahemad M., Khan M.S. (2011). Functional aspects of plant growth promoting rhizobacteria: recent advancements. Insight Microbiol.

[166] Kavamura V.N., Santos S.N., Silva J.L., Parma M.M., Ávila L.A. (2013). Screening of Brazilian cacti rhizobacteria for plant growth promotion under drought. Microbiol Res.

[167] García-Jiménez B., Torres-Bacete J., Nogales J. (2021). Metabolic modelling approaches for describing and engineering microbial communities. Comput Struct Biotechnol J..

